# Microalgae and microbial inoculant as partial substitutes for chemical fertilizer enhance *Polygala tenuifolia* yield and quality by improving soil microorganisms

**DOI:** 10.3389/fpls.2024.1499966

**Published:** 2025-01-16

**Authors:** Yuying Su, Ying Ren, Gang Wang, Jinfeng Li, Hui Zhang, Yumeng Yang, Xiaohui Pang, Jianping Han

**Affiliations:** Institute of Medicinal Plant Development, Chinese Academy of Medical Sciences & Peking Union Medical College, Beijing, China

**Keywords:** chemical fertilizer reduction, bio-organic fertilizer, rhizosphere microorganism, medicinal plant cultivation, *Polygala tenuifolia*

## Abstract

Excessive utilization of chemical fertilizers degrades the quality of medicinal plants and soil. Bio-organic fertilizers (BOFs) including microbial inoculants and microalgae have garnered considerable attention as potential substitutes for chemical fertilizer to enhance yield. In this study, a field experiment was conducted to investigate the effects of BOF partially substituting chemical fertilizer on the growth and quality of medicinal plant *Polygala tenuifolia*. The growth parameters, bioactive component contents, soil properties and composition of rhizosphere microorganisms were measured. The results indicated that substituting 40% of chemical fertilizer with microalgae showed the most pronounced growth-promoting effect, leading to a 29.30% increase in underground biomass and a 19.72% increase in 3,6’-disinapoylsucrose (DISS) content. Substituting 20% of chemical fertilizer with microalgae improved soil quality, significantly increasing soil organic matter content by 15.68% (*p*<0.05). Microalgae addition significantly affected the rhizosphere bacterial community composition of *P. tenuifolia*, reducing the relative abundance of *Cladosporium* by 33.33% and 57.93%, while increasing the relative abundance of Chloroflexi by 31.06% and 38.27%, under 20% and 40% chemical fertilizer reduction, respectively. The relative abundance of Chloroflexi positively correlated with both the underground biomass and DISS content (*p*<0.05), indicating that microalgae may stimulate Chloroflexi species associated with carbon cycling, thereby enhancing soil fertility, nutrient absorption, and ultimately leading to increased biomass accumulation and production of bioactive components in *P. tenuifolia*. In addition, there was no significant difference in underground growth and bioactive component contents between reduced chemical fertilizer dosage combined with solid microbial inoculant (SMI) and polyglutamic microbial inoculant (PMI), compared with 100% chemical fertilizer. Correlation analysis revealed that PMI could increase soil phosphorus availability through *Streptomyces* recruitment. In conclusion, our findings demonstrated that bio-organic fertilizers can partially substitute chemical fertilizer to improve soil properties and microorganisms, enhancing the growth and quality of *P. tenuifolia*. This provides a theoretical basis for increasing medicinal plant productivity under chemical fertilizer reduction.

## 1 Introduction

*Polygala tenuifolia*, which uses the roots for medicinal purposes, is commonly employed for treating insomnia, memory disorders, and neurosis. This perennial medicinal plant holds potential as a preventive and therapeutic agent against Alzheimer’s disease, leading to high market demands (Deng et al., 2020; Zhang et al., 2023). The increasing demand for *P. tenuifolia* has promoted a drive for higher yields, thus resulting in the overuse of agrochemicals. However, it was reported that over 50% of nitrogen in fertilizers applied cannot be absorbed by crops (Saud et al., 2022). On the other hand, agrochemical overuse may lead to a decline in soil organic matter content, as well as soil microbial diversity and abundance, result in soil compaction, acidification, and imbalanced nutrient structure, ultimately compromising both the quality of *P. tenuifolia* and soil health (Guo et al., 2010; Liu et al., 2020; Zhang et al., 2020). Therefore, it is necessary to reduce chemical fertilizer usage and improve utilization rate. Currently in agriculture, there is a conscious effort being made to develop and utilize bio-organic fertilizers (BOFs) as substitutes for chemical fertilizers and pesticides to reduce harm to crops and soil (Waltz, 2017).

Microalgae fertilizers (MFs) and microbial inoculants (MIs) have been commercially available as BOFs, biopesticides and soil amendments (Renuka et al., 2018; Klimasmith and Kent, 2022; Parmar et al., 2023). MIs contain single or multiple strains of live or dormant microorganisms, including plant growth-promoting rhizobacteria (PGPRs) and beneficial fungi, such as *Azotobacter* sp., *Azospirillum* sp., *Bacillus* sp., *Rhizobium* sp., *Sphingobium* sp., *Streptomyces* sp., and *Pseudomonas* sp., which could interact with plants and exert conducive influence on the composition and structure of soil microbial communities (Zhuang et al., 2007; Berendsen et al., 2012; Ambrosini et al., 2016; Sousa et al., 2016). *Bacillus* sp., such as *Bacillus amyloliquefaciens*, *B. subtilis*, and *B. mucilaginosus*, are PGPRs commonly used in bio-fertilizers, and also constitute the primary constituents of the MIs utilized in this study (Abou-El-Seoud and Abdel-Megeed, 2012; Sun et al., 2020; Xue et al., 2021). The underlying mechanisms responsible for their growth-promoting effects can be summarized into the following three points: (1) improving plant nutrient utilization through nitrogen fixation and phosphorus solubilization (Klimasmith and Kent, 2022); (2) producing plant growth hormones and enzymes (Trabelsi and Mhamdi, 2013); (3) providing biocontrol against filamentous fungi and pathogens (Cao et al., 2011; Wang et al., 2021; Deng et al., 2022; Luo et al., 2022). Microalgae primarily promote plant growth and enhance soil fertility through nitrogen fix, carbon sequester through photosynthesis, and synthesis of metabolites (Marks et al., 2017; Abinandan et al., 2019; Sido et al., 2022). Additionally, PGPRs and nitrogen-fixing cyanobacteria can also assist in phytoremediation by removing toxic metals and organic pollutants from soil and fostering a more ordered and efficient microbial community (Kong et al., 2019; Rezasoltani and Champagne, 2023). Both *Bacillus* MI and microalgae can reduce NO and N_2_O emissions or sequester CO_2_ from the atmosphere, thereby mitigating the greenhouse effect, global warming, and climate change (Calvo et al., 2013; Cheah et al., 2015; Calvo et al., 2016; Wu et al., 2018).

The combination of bio-organic fertilizer and chemical fertilizer has been found to result in superior crop yield and quality compared to using only bio-organic fertilizer (Zhang et al., 2024). As mentioned earlier, both MIs and MFs have the potential to enhance plant nutrient uptake and reduce nitrogen loss, thus increasing the efficiency of chemical fertilizer utilization (Xue et al., 2021). Studies have demonstrated that the co-application of *Bacillus* MIs or microalgae with chemical fertilizer can regulate the structure of soil microbial communities to achieve coordination and supplementation of chemical fertilizer, leading to enhanced soil nutrient content and ultimately increasing crop yield (Cao et al., 2023). Under deficit irrigation regimes, reducing chemical fertilizer application in conjunction with MI can enhance fenugreek yield while minimizing chemical fertilizer consumption (Dadrasan et al., 2015). Microalgae biomass grown in domestic wastewater can be partially substituted for chemical fertilizer in the cultivation of basil crops (*Ocimum basilicum* L.) (Álvarez-González et al., 2022). It is noteworthy that BOFs can serve as a viable strategy to maintain or improve crop yields. However, insufficient application of chemical fertilizer or sole reliance on BOF may result in yield reduction (Ye et al., 2020). Studies have shown that a substitution rate of 20%-40% with chemical fertilizer is more beneficial in promoting nutrient absorption and increasing crop yields, while reducing environmental pollution and improving soil microenvironment (Adesemoye et al., 2009; Lei et al., 2012; Jin et al., 2022; Yuan et al., 2023). Therefore, BOFs hold promising potential in partially substituting agrochemicals for sustainable crop and medicinal plant production.

However, the current research has primarily focused on the application of BOFs in crop production, with limited studies conducted on their effects in medicinal plants (Renuka et al., 2018; Shahwar et al., 2023). Medicinal plants, particularly, differ from general crops as their quality directly impacts efficacy and safety. Therefore, the objectives of this study were (1) to investigate the effects of chemical fertilizer reduction and application of different bio-organic fertilizers on the growth and quality of *P. tenuifolia*, and (2) to explore the mechanism of bio-organic fertilizers partially substituting chemical fertilizer by analyzing soil properties and microorganisms, in order to provide theoretical basis for eco-friendly fertilizer applications in medicinal plant cultivation.

## 2 Materials and methods

### 2.1 Experimental sites

Field experiment was conducted in Wenxi County, Yuncheng City, Shanxi Province (111°13’27’’ E, 35°26’26’’ N), the primary production region for *Polygala tenuifolia*. A biennial *P. tenuifolia* field with consistent growth was selected. The cultivated *P. tenuifolia* was identified by Professor Jianping Han of the Institute of Medicinal Plant Development, Chinese Academy of Medical Sciences & Peking Union Medical College following the standards outlined in the Pharmacopoeia of the People’s Republic of China (PPRC-2020). The specimens were preserved at the same institution. The region has a temperate continental monsoon climate, with an average annual temperature of 12.5 °C. The annual precipitation is 506 mm, and this region is susceptible to drought. The tested soil type is sandy loam soil. The plowing layer soil (0-20 cm) had a pH of 8.11, an organic matter content of 15.80 g/kg, a total nitrogen content of 1.08 g/kg, a total phosphorus content of 0.59 g/kg, a total potassium content of 25.70 g/kg, an alkali-hydrolyzable nitrogen content of 74.19 mg/kg, an available phosphorus content of 11.50 mg/kg, and an available potassium content of 215.00 mg/kg.

### 2.2 Experimental treatments and maintenance

Seven treatments were established as shown in **Table 1**: (1) CF: 100% chemical fertilizer; (2) PMI1: -20% chemical fertilizer + polyglutamic acid microbial inoculant; (3) PMI2: -40% chemical fertilizer + polyglutamic acid microbial inoculant; (4) SMI1: -20% chemical fertilizer + solid microbial inoculant; (5) SMI2: -40% chemical fertilizer + solid microbial inoculant; (6) MF1: -20% chemical fertilizer + microalgae fertilizer; (7) MF2: -40% chemical fertilizer + microalgae fertilizer. The chemical fertilizer (N: P: K = 16: 5: 24) was purchased from Liuguo Chemical Co. Ltd (Anhui, China) with an application rate of 750 kg/ha. The application rate of chemical fertilizer used in treatments with a 20% and 40% reduction in chemical fertilizer is 600 kg/ha and 450 kg/ha, respectively. The Gulefeng 8.8 billion^®^ polyglutamic acid microbial inoculant (PMI, viable count ≥ 8.8×10^9^/mL) was purchased from Xuankai Biotechnology (Xuankai Biotechnology Co. Ltd., Nanjing, China) and utilized in accordance with the manufacturer’s instructions, at a dosage of 75L diluted microbial inoculant per hectare (diluted at a ratio of 1:100). PMI primarily consists of polyglutamic acid (PGA), *Bacillus amyloliquefaciens*, *B. subtilis* and *Brevibacillus laterosporu*. The solid microbial inoculant (SMI, viable count ≥ 2.0×10^8^/g) was produced by Jintu Biotechnology (Jintu Biotechnology Co. Ltd., Hebei, China) and utilized at a dosage of 150 kg/ha. Its main components are *Bacillus amyloliquefaciens* and *Brevibacillus laterosporu*. The Titubang^®^ microalgae fertilizer (MF, viable count ≥ 1.0×10^5^/mL) was purchased from Bailing Biotechnology (Bailing Biotechnology Co. Ltd., Beijing, China) and utilized at a dosage of 50 L diluted microbial inoculant per hectare (diluted at a ratio of 1:100). *Chlorella pyrenoidosa*, nitrogen-fixing cyanobacteria and *Tolypothrix tenuis* are the main components. Each treatment consisted of three biological replicates, with each replicate covering an area of 12 m^2^ (2 m×6 m). To minimize the marginal effect, protection lines (0.5 m) were placed between each replicate cell. The experiment was initiated in April 2023 with the first application of biennial *P. tenuifolia*, followed by subsequent applications of the same dosage of BOFs administered every two months, for a total of three times.

**Table 1:** Details of the treatments used in the field experiment.

Treatment	Chemical fertilizer application rate	N/P/K input	Bio-organic fertilizer application rate	Application method
CF	750 kg/ha	≥120/37.5/180 kg/ha	–	Furrow application
PMI1	600 kg/ha	≥96/30/144 kg/ha	75 L/ha	Root irrigation
PMI2	450 kg/ha	≥72/22.5/108 kg/ha	75 L/ha	Root irrigation
SMI1	600 kg/ha	≥96/30/144 kg/ha	150 kg/ha	Furrow application
SMI2	450 kg/ha	≥72/22.5/108 kg/ha	150 kg/ha	Furrow application
MF1	600 kg/ha	≥96/30/144 kg/ha	50 L/ha	Root irrigation
MF2	450 kg/ha	≥72/22.5/108 kg/ha	50 L/ha	Root irrigation

### 2.3 Determination of underground biomass and quality of *P. tenuifolia*

After six months, *P. tenuifolia* were sampled, and plant traits were collected from at least ten plants in each biological replicate. The underground biomass of each *P. tenuifolia* root was determined, while the root diameter was measured using vernier scale. All root samples were processed by removing the woody core (xylem), dried at 55 °C, powdered, and sieved through a 50-mesh sieve. The contents of polygalaxanthone III (POL) and 3,6’-disinapoylsucrose (DISS) in *P. tenuifolia* were determined by high-performance liquid chromatography (HPLC). 0.5 g of *P. tenuifolia* powder was mixed with 10 mL of 70% methanol, weighed, and subjected to ultrasonic extraction (400 W, 40 kHz) for 45 min. After cooling and re-weighing, any weight loss was compensated by adding additional 70% methanol. The mixture was centrifuged at a speed of 5000 r/min for 5 min, and the supernatant was filtered through a 0.22 μm filter membrane. A Shimadzu LC-2030 HPLC system (Shimadzu, Kyoto, Japan) equipped with an Agilent ZORBAX Extend-C18 column (46×250 mm, 5 μm) was employed for isocratic elution. The mobile phase consisted of acetonitrile and 0.05% phosphoric acid solution at a ratio of 18:82 (*v*/*v*) with a flow rate of 1.0 mL/min. Detection was conducted at a wavelength of 320 nm, while the column temperature was 30 °C and the injection volume was 10 μL. A series of working standard solutions were set up with a mixed standard solution comprising POL and DISS, both of which were purchased from Yuanye Biotechnology Co., Ltd (Shanghai, China).

### 2.4 Soil sampling and determination

When harvesting *P. tenuifolia* plants, rhizosphere soil tightly adhering to the root surface were collected. One sample of *P. tenuifolia* rhizosphere soil was collected per replicate, with three replicates per treatment. Each sample represented a composite of soil collected from the rhizosphere of plants within its respective replicate plot. All rhizosphere soil samples were sieved and stored at -80 °C for microbial analysis. In each replicated plot, bulk soil samples weighing approximately 500 g were collected using a five-point sampling method at depths ranging from 0 to 20 cm around the plants. Three replicates were taken for each treatment. The collected soil samples were air-dried, sieved through a 100-mesh sieve, and subjected to analysis for pH and the contents of soil organic matter (SOM), alkali-hydrolyzable nitrogen (AN), available potassium (AK) and available phosphorus (AP). The soil pH was tested by the potentiometric method, while the organic matter content was determined using the K_2_CrO_7_/H_2_SO_4_ oxidation method (Marks et al., 2017; Shrestha et al., 2022). The alkaline hydrolysis diffusion method was employed to determine the soil alkali-hydrolyzable nitrogen content (Wang et al., 2022). The molybdenum antimony anti-colorimetric method was used to measure the soil available phosphorus content (Chu and Grogan, 2010). The flame spectrophotometer technique was utilized to assess the soil available potassium content (Li et al., 2022).

### 2.5 Sequencing

Total genome DNA from all rhizosphere soil samples were extracted using CTAB method (Kamdem et al., 2023; Zheng et al., 2024). DNA concentration and purity was monitored on 1% agarose gels. The 16S rRNA gene of bacteria was amplified using the primers 341F (5’-CCTAYGGGRBGCASCAG-3’)/806R (5’-GGACTACNNGGGTATCTAAT-3’), and the ITS region of the fungi was amplified with ITS1F (5’-CTTGGTCATTTAGAGGAAGTAA-3’)/ITS2R (5’-GCTGCGTTCTTCATCGATGC-3’) primers (Wei et al., 2021). The polymerase chain reaction (PCR) system was composed as follows: 15 μL of Phusion^®^ High – Fidelity PCR Master Mix (New England Biolabs), 0.2 μM of forward and reverse primers, and about 10 ng template DNA. Thermal cycling consisted of initial denaturation at 98°C for 1 min, followed by 30 cycles of denaturation at 98°C for 10 s, annealing at 50°C for 30 s, elongation at 72°C for 30 s; then ended with an extension at 72°C for 5 min. The integrity and concentration of PCR products were verified by 2% agarose gel electrophoresis. PCR products were purified with Qiagen Gel Extraction Kit (Qiagen, Germany). Sequencing libraries were generated using TruSeq^®^ DNA PCR-Free Sample Preparation Kit (Illumina, USA) and index codes were added. The library quality was assessed on the Qubit@ 2.0 Fluorometer (Thermo Scientific) and Agilent Bioanalyzer 2100 system. At last, the library was sequenced on an Illumina NovaSeq platform by Novogene Co., Ltd (Beijing, China) and 250 bp paired-end reads were generated.

### 2.6 Bioinformatics analysis

Paired-end reads were first assigned to samples based on unique barcodes, then trimmed to remove barcode and primer sequences, and merged using FLASH. Quality filtering was done with Fastp (v. 0.23.1) to obtain high-quality clean tags. These tags were compared against the Silva database (16S) and Unite database (ITS) using the UCHIME Algorithm to detect and remove chimera sequences, resulting in effective tags. Denoising was then performed using the DADA2 module in QIIME2 (Version QIIME2-202202) to obtain initial amplicon sequence variants (ASVs). Species annotation was conducted using the Silva database (16S) and Unite database (ITS) within QIIME2. Multiple sequence alignment was done using QIIME2 to investigate phylogenetic relationships and differences in predominant species among sample groups. The absolute abundance of ASV was normalized based on the sample with the fewest sequences. Subsequent alpha and beta diversity analyses were performed using the normalized data.

GraphPad Prism (V10.0.3) was used to plot histograms representing the average values of the parameters, with bars indicating the standard error of the mean (SEM). The statistical differences were analyzed using one-way analysis of variance (ANOVA) with SPSS V26.0 statistical software (IBM, USA). Alpha diversity indices were measured through QIIME2 to analyze the diversity and richness. Principal coordinate analysis (PCoA) of rhizosphere microbial community composition were performed using Bray-Curtis distance. Biomarkers were identified by the Linear discriminant analysis effect size (LefSe) with a linear discriminant analysis (LDA) score > 2.0 and *p* < 0.05. Redundancy analysis (RDA) was used to explore the relationships between soil properties and microbial composition using R (V4.3.1). R (V4.3.1) was also used to estimate the correlation among plant growth indicators, quality indicators, soil properties and rhizosphere microbial communities by Pearson and Mantel correlation analysis.

## 3 Results and discussion

### 3.1 *P. tenuifolia* growth

**Figure 1:**
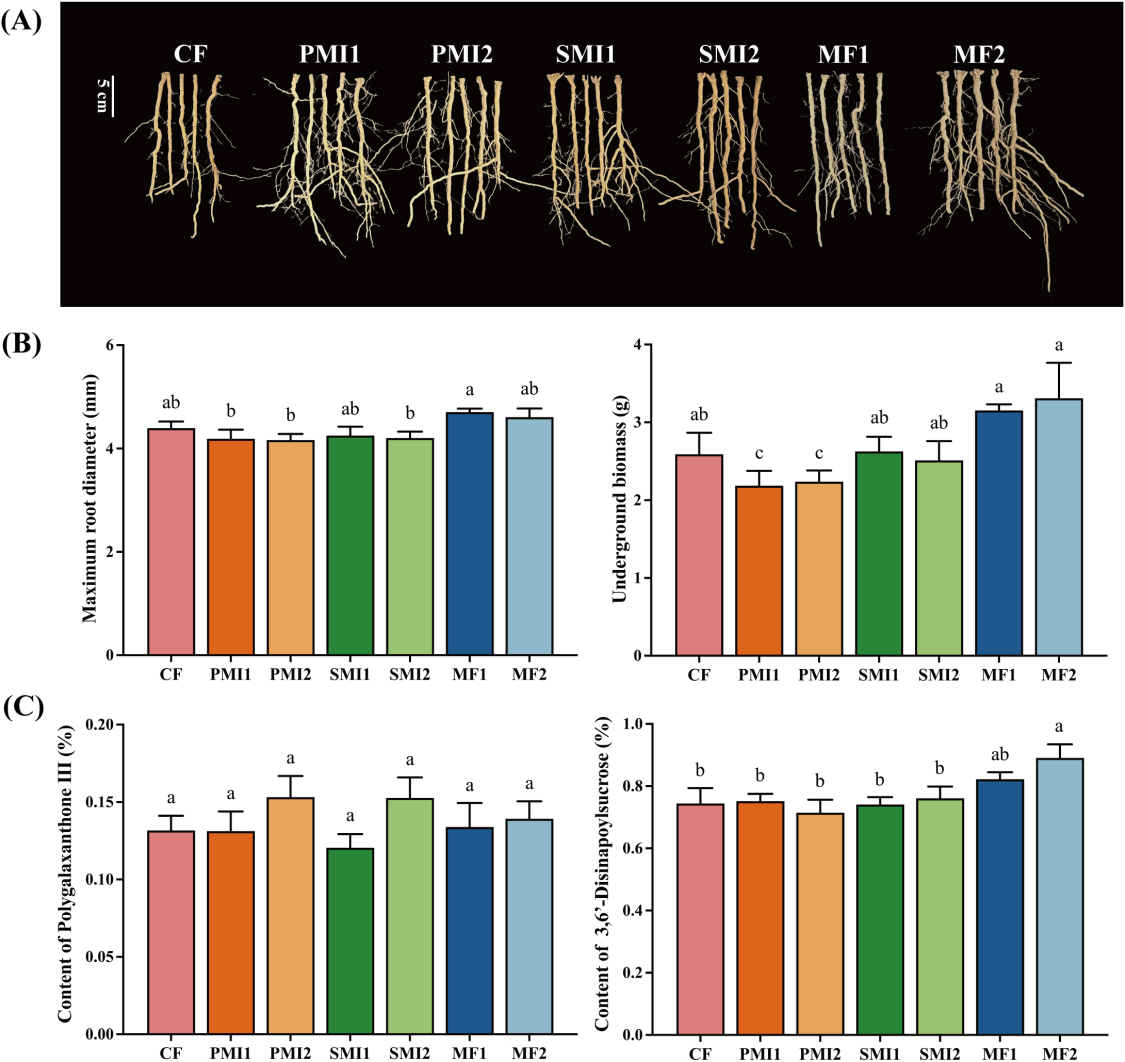
Effect of partial substitution of BOFs for chemical fertilizer on root phenotype **(A)**, underground growth **(B)** and bioactive component contents **(C)**. CF, 100% chemical fertilizer; PMI1, -20% chemical fertilizer + polyglutamic acid microbial inoculant; PMI2, -40% chemical fertilizer + polyglutamic acid microbial inoculant; SMI1, -20% chemical fertilizer + solid microbial inoculant; SMI2, -40% chemical fertilizer + solid microbial inoculant; MF1, -20% chemical fertilizer + microalgae fertilizer; MF2, -40% chemical fertilizer + microalgae fertilizer. The different lowercase letters indicated the significant difference among the different groups at p<0.05 level.

In this study, combined application of reduced chemical fertilizer and BOF increased *P. tenuifolia* underground growth traits including underground biomass and root diameter (**Figure 1**). MF showed the greatest efficacy in promoting both underground biomass and root diameter of *P. tenuifolia* (**Figure 1B**). The underground biomass of *P. tenuifolia* increased by 23.05% and 29.30% under MF1 and MF2 respectively, while the root diameter experienced respective increments of 6.82% and 4.78%. Reducing the application of chemical fertilizer and combining it with SMI and PMI did not result in a significant difference in the underground growth of *P. tenuifolia* when compared to applying 100% chemical fertilizer.

Microbial inoculants with *Bacillus* as the core microorganism exert a significant effect on increasing production (Chen et al., 2021; Shi et al., 2022). In this study, the underground biomass of *P. tenuifolia* in PMI and SMI groups were comparable to CF under reduced chemical fertilizer usage conditions, indicating that MIs partially substituting chemical fertilizer can ensure efficient production (**Figure 1B**). This effect may be attributed to the nitrogen fixation and phosphorus solubilization functions of the main functional bacteria including *Bacillus subtilis*, *Bacillus amyloliquefaciens*, and *Bacillus mucilaginosus* added in MIs, as well as their abilities to release growth-promoting hormones. Furthermore, poly-γ-glutamic (γ-PGA), a novel fertilizer synergist and one of the main components in PMI, not only exhibits remarkable water and fertilizer retention abilities but also enhances plant nutrient absorption by increasing root biomass and activity (Yin et al., 2018; Bai et al., 2022). Researchers had also discovered that the combination of microalgae with organic fertilizers or their partial substitution for chemical fertilizer can improve crop yield (Álvarez-González et al., 2022; Cao et al., 2023). This study confirmed that among different BOFs applied in this study, co-application of microalgae and chemical fertilizer has the best promotion effects on *P. tenuifolia* growth compared to 100% chemical fertilizer group (**Figure 1B**). Additionally, as shown in **Figure 1A**, MF significantly enhanced the growth of lateral and fibrous roots. This may be attributed to the pivotal role of indole-3-acetic acid (IAA) produced by various microalgae in primary and lateral root development (Zhang et al., 2024). Overall, partial substitution of chemical fertilizer with BOF provided a feasible strategy to reduce chemical fertilizer usage, thereby promoting underground growth of *P. tenuifolia* and increasing yield.

### 3.2 Bioactive component contents

As shown in **Figure 1C**, MF significantly increased the content of DISS in the roots of *P. tenuifolia*, MF1 and MF2 increased the content of DISS by 10.45% and 19.72%, respectively, with the increase in MF2 being statistically significant (*p* < 0.05). While other BOFs treatments as partial substitutes for chemical fertilizer achieved comparable effectiveness to 100% chemical fertilizer in terms of DISS and POL contents in *P. tenuifolia*.

POL has anti-inflammatory properties, and DISS is known for its significant anxiolytic and antidepressant effects (Hu et al., 2011; Yang et al., 2022; Zhang et al., 2022). They are major bioactive components of *P. tenuifolia*, playing a crucial role in determining the clinical efficacy of *P. tenuifolia* roots, which serve as important quality control indicators (Xu et al., 2016). The content of oligosaccharide esters including DISS in *P. tenuifolia* is significantly influenced by soil type and origin, while there is minimal variation in xanthones (Pu et al., 2017; Ji et al., 2023). This suggested a close relationship between the accumulation of DISS during the growth process of *P. tenuifolia* and environmental factors such as climate, soil properties, and rhizosphere microbial communities. These findings supported our experimental results and confirmed that the application of MF may create more favorable conditions for DISS accumulation in *P. tenuifolia*. Our previous investigations have demonstrated that *Bacillus* sp. can facilitate the accumulation of total tanshinone in *Salvia miltiorrhiza* (Wei et al., 2023). Furthermore, many studies had shown that MF can increase the nutrient contents of crops, vegetables and fruits, including soluble sugars, vitamin C, protein, chlorophyll and carotenoids (Coppens et al., 2016, 2016; Zhang et al., 2024). MF also has the ability to increase the content of secondary metabolites in plants (Díaz et al., 2024). For instance, applying *Coelastrella* sp. can elevate most organic acids and phenolic compounds in strawberries by more than 10% (Žunić et al., 2024). The above results demonstrated that microalgae could improve plant quality while promoting plant growth, indicating its potential for further study and application in the cultivation of medicinal plants (**Figure 1C**). Meanwhile, based on the results of this study, further research is needed to investigate the mechanism by which microalgae promote DISS accumulation in *P. tenuifolia*.

### 3.3 Soil properties

**Figure 2:**
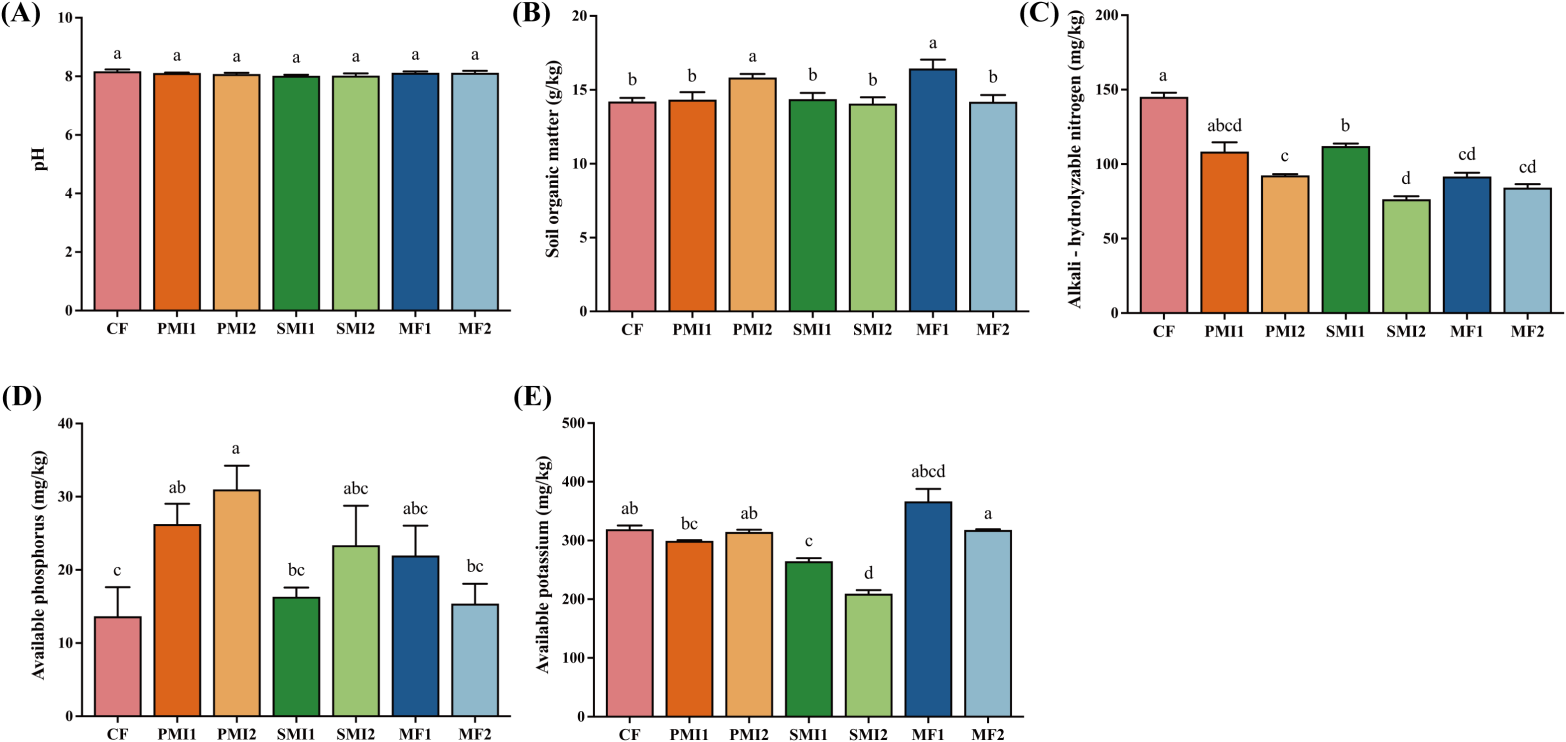
Soil physicochemical properties under different fertilization treatments. **(A)** pH; **(B)** soil organic matter; **(C)** alkali-hydrolyzable nitrogen; **(D)** available phosphorus; **(E)** available potassium. CF, PMI1, PMI2, SMI1, SMI2, MF1, and MF2 were as defined in the footnote to **Figure 1**. The different lowercase letters indicated the significant difference among the different groups at p<0.05 level.

As shown in **Figure 2**, the partial substitution of chemical fertilizer with BOFs had a significant impact on soil physicochemical properties. PMI2 and MF1 exhibited a substantial increase in SOM by 11.32% and 15.68%, respectively (*p* < 0.05). CF group displayed the highest AN content, while soils treated with 80% chemical fertilizer application demonstrated higher AN level than those treated with 60% chemical fertilizer application, suggesting that inorganic nutrients provided by chemical fertilizer are primarily responsible for increasing inorganic nitrogen content in the soil. All the six treatments with BOFs showed a higher availability of P in soils as compared to CF. Among them, the AP content processed by PMI1 and PMI2 is 1.92 times and 2.27 times that of CF, respectively (*p* < 0.05).

In addition to the reduction in chemical fertilizer usage, the decrease of AN may also be attributed to the incorporation of BOFs, which enhanced nitrogen absorption by *P. tenuifolia* (Zhou et al., 2023). Beneficial microorganisms present in MIs can effectively colonize soils and secrete organic acids that dissolve and release nitrogen nutrients adsorbed onto soil particles, while also modifying indigenous bacterial communities involved in nitrogen cycling (van der Heijden et al., 2008; Lei et al., 2012; Huang et al., 2022). Application of microalgae can enhance soil enzyme activity and promote life activities of microorganisms related to nitrogen cycling, while nitrogen-fixing cyanobacteria can provide fixed-nitrogen through biological fixation or conversion between different forms of nitrogen fertilizers (Renuka et al., 2018; Zhou et al., 2023). Hence, the synergistic application of chemical fertilizer and MIs confers greater advantages on nitrogen uptake by plants compared to their individual applications. In addition to N nutrition, the inoculation of BOFs can augment the availability and translocation of various micro- and macronutrients, including Zn, Cu, Fe, C, P, K within the soils and plants (Coppens et al., 2016; Renuka et al., 2018). Various BOFs including *Bacillus* sp. and microalgae have been found to possess phosphate solubilization ability, which can lower soil pH and convert soil-bound phosphorus into soluble forms that are readily accessible for plant growth. This transformation is also facilitated by metabolites released during microbial metabolism and low molecular weight organic acids. The partial substitution of chemical fertilizer with BOFs primarily stimulate plant growth through microbial activities and interactions with soil or plants rather than directly supplying various nutrients to the soil. Consequently, changes in properties of the soil may be influenced by factors such as fertilization amount, fertilizer ratio, and duration of application. In conclusion, the partial substitution of chemical fertilizer with BOFs have shown potential to enhance nitrogen nutrient uptake by *P. tenuifolia* in soil and can significantly improve the availability of phosphorus, which is closely related to the beneficial effects of microorganisms.

### 3.4 Rhizosphere microbial community

#### 3.4.1 Rhizosphere microbial diversity of *P. tenuifolia*

**Figure 3:**
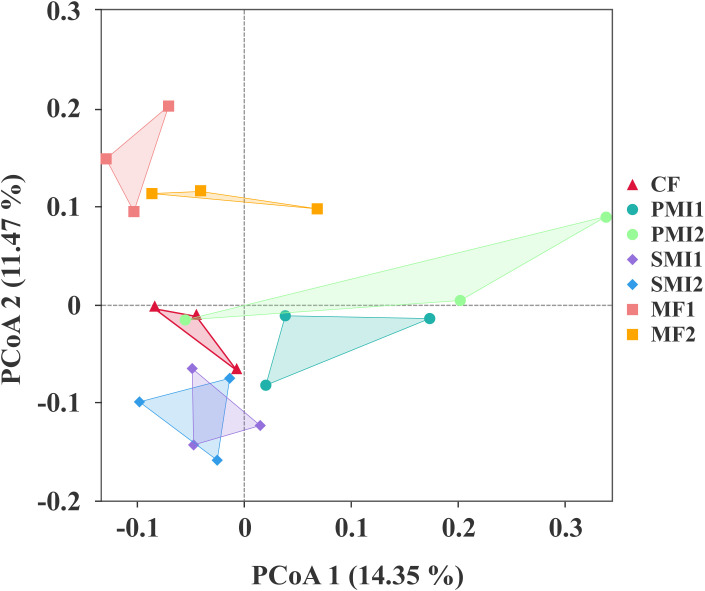
Principal coordinate analysis (PCoA) plot based on the rhizosphere bacterial community composition. CF, PMI1, PMI2, SMI1, SMI2, MF1, and MF2 were as defined in the footnote to **Figure 1**.

After quality control filtering, a total of 20,178 bacterial ASVs and 2,995 fungal ASVs were detected. PCoA analysis based on rhizosphere bacterial community composition revealed that samples within the same BOF treatment groups clustered together and MF groups showed distinct separation from other groups (**Figure 3**). This indicated that the addition of MF significantly influenced bacterial community composition. Furthermore, a more pronounced impact observed on bacterial communities compared to fungal communities (**Supplementary Figure S1**). The application of BOFs may altered soil environmental conditions and promoted an enrichment of specific functional microbial groups within the rhizosphere, thereby diminishing species biodiversity (Yang et al., 2022). Additionally, the introduction of exogenous microorganisms can potentially disturb native microbial communities, resulting in either antagonistic or synergistic effects (Qiu et al., 2009; Ambrosini et al., 2016). It should also be noted that the influence exerted by external additions like BOFs on soil microflora has strong temporal dependency (Nguyen et al., 2018).

#### 3.4.2 Composition of the rhizosphere bacterial community

**Figure 4:**
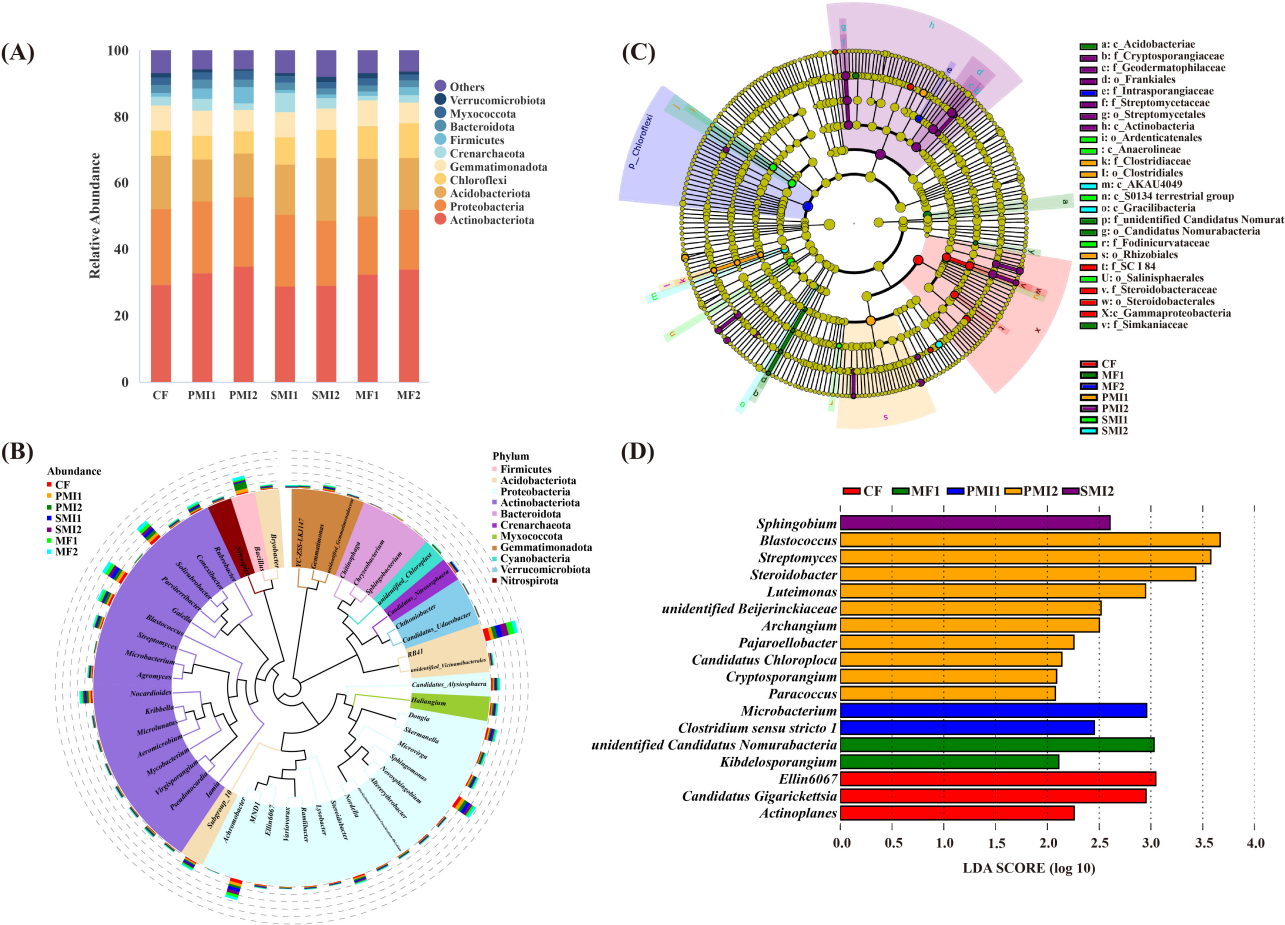
Composition of rhizosphere bacterial communities in *P. tenuifolia*. **(A)** The relative abundance of top 10 bacterial phyla. **(B)** Phylogenetic tree of top 50 bacterial genera based on relative abundance. **(C)** Significantly enriched bacterial taxa showed by cladograms based on linear discriminant (LEfSe) analysis. **(D)** Scores for the bacterial genera showed by bar chart based on LEfSe analysis. CF, PMI1, PMI2, SMI1, SMI2, MF1, and MF2 were as defined in the footnote to **Figure 1**.

The clustering results revealed that the bacterial communities of different fertilization treatments belonged to 41 phyla, 98 classes, 216 orders, 298 families and 547 genera based on the detected ASVs. At each taxonomic level, there were certain ASVs that remain unidentified. Actinobacteriota, Proteobacteria, Acidobacteriota, Chloroflexi and Gemmatimonadota were identified as the dominant phyla with a combined relative abundance (RA) exceeding 80% in the bacterial community (**Figure 4A**), which was consistent with previous study (Gu et al., 2023). Compared to CF, both SMI and MF groups showed an increased RA of Chloroflexi. Therefore, it can be concluded that the application of SMI and MF alleviated the decline in Chloroflexi caused by chemical fertilizer.

The predominant genera were identified as *RB41* (RA: 2.12-4.06%), *Sphingomonas* (1.32-2.35%), *MND1* (1.36-2.39%), *Solirubrobacter* (1.73-2.30%), and *Gaiella* (1.84-2.24%) (**Figure 4B**). The PMIs groups showed the greatest promotional effect in the RA of *Bacillus*. This could be attributed to the substantial proliferation and recruitment of *Bacillus* in soil after their application. Unexpectedly, the RA of *Bacillus* in SMIs groups was found to be lower compared to that in CF. It is possible that *Bacillus*, as endophytes colonizing within *P. tenuifolia* plants, negatively affected the RA of rhizosphere *Bacillus* (Wang et al., 2020). Another plausible explanation arised from differences in fertilization methods. Bacterial movement between ecological niches primarily relies on rainfall. In this experiment, both PMI and MF were applied through spraying techniques; however, SMI employed furrow application due to its solid-state fertilizer nature. Despite timely watering after fertilization, this application method might hinder microorganisms from transferring effectively from fertilizer into soil, thereby potentially contributing to a low survival rate of exogenous microbial communities (Semenov et al., 2021). The result indicated that *Streptomyces* significantly increased in PMI2 (*p*<0.05). A limited number of bacterial genera, including *Streptomyces*, were primarily responsible for the release of carbon dioxide from soil (Stone et al., 2021). Moreover, *Streptomyces* contribute to enhanced resistance against biotic and abiotic stresses in plants. Apart from acting as biocontrol agents through antibiotic production, *Streptomyces* promotes plant growth by solubilizing phosphorus and producing plant growth hormone-like substances such as IAA (Kim et al., 2011; Vurukonda et al., 2018; Omar et al., 2022). In summary, the partial substitution of chemical fertilizer with BOFs can alter the composition of rhizosphere bacterial communities and increase the RAs of plant growth-promoting bacteria.

According to the LEfSe analysis, the application of BOFs caused significant changes in specific taxonomic groups, thereby exerting a profound influence on rhizosphere ecological functions (**Figures 4C, D**). At the genus level, functional classification of specific biomarkers in each treatment revealed that BOFs can enrich beneficial bacteria capable of promoting plant growth and participating in soil bioremediation. Firstly, PGPRs were enriched. Samples of the PMI2 group detected the highest abundance of *Streptomyces*. *Kibdelosporangium* was the biomarker of MF1, which possesses 1-aminocyclopropane-1-carboxylate deaminase acid (ACCD) activity, enabling it to reduce ethylene concentration in plants under stress conditions, improve plant stress resistance, and thereby promote the growth of host plants (Xing et al., 2012; Qin et al., 2015; Ali and Kim, 2018; Xiong et al., 2019). *Microbacterium* and *Sphingobium*, which possess soil bioremediation capabilities, were significantly enriched in PMI1 and SMI2, respectively. *Microbacterium* sp. is capable of producing plant hormones such as IAA, which can remediate heavy metal pollution and enhance plant growth under multi-heavy metal stress (Ren et al., 2019; Sun et al., 2019). Furthermore, the volatiles produced by root-associated bacteria of *Microbacterium* can promote plant growth without requiring direct and prolonged contact with plants, which is likely achieved through the regulation of sulfur and nitrogen metabolism (Cordovez et al., 2018; Liu et al., 2022). There are multiple PGPRs present in *Sphingobium*, which exhibit resistance to heavy metals and possess the ability to degrade aromatic pollutants (Thomas et al., 2019; Boss et al., 2022; Liu et al., 2023; Zou et al., 2023). Overall, both MIs and MFs can optimize rhizosphere bacterial communities by regulating soil microbial activity and recruiting agricultural beneficial microorganisms (Xiao and Zheng, 2016; Wang et al., 2021).

#### 3.4.3 Composition of the rhizosphere fungal community

**Figure 5:**
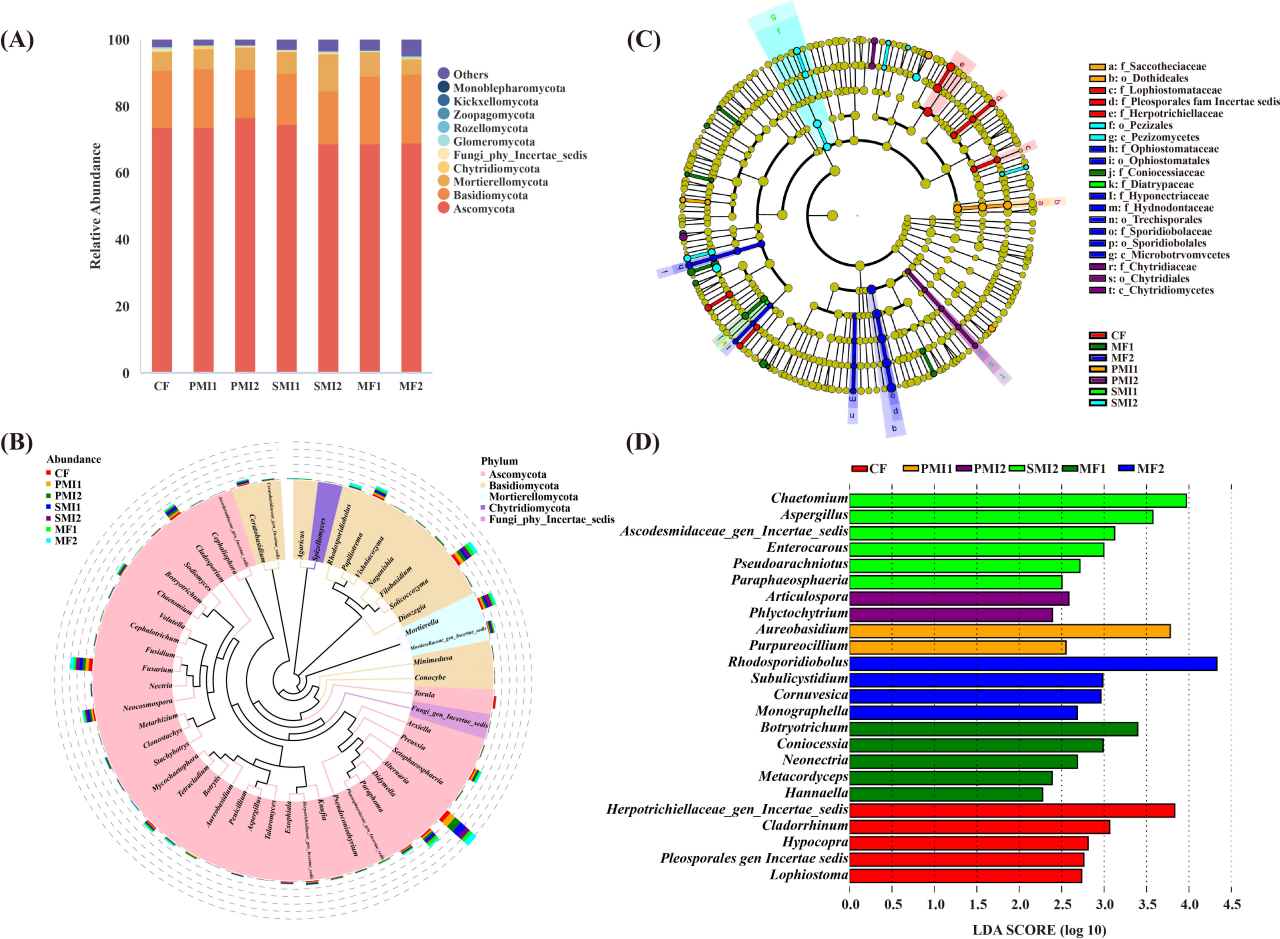
Composition of rhizosphere fungal communities in *P. tenuifolia*. **(A)** The relative abundance of top 10 fungal phyla. **(B)** Phylogenetic tree of top 50 fungal genera based on relative abundance. **(C)** Significantly enriched fungal taxa showed by cladograms based on linear discriminant (LEfSe) analysis. **(D)** Scores for the fungal genera showed by bar chart based on LEfSe analysis. CF, PMI1, PMI2, SMI1, SMI2, MF1, and MF2 were as defined in the footnote to **Figure 1**.

The fungal community of *P. tenuifolia* rhizosphere is taxonomically classified into 13 phyla, 46 classes, 83 orders, 179 families and 350 genera. At each taxonomic level, there were some ASVs that cannot be identified. The combined of Ascomycota (RA: 68.83%-76.72%), Basidiomycota (14.42%-20.78%), and Mortierellomycota (4.46%-11.24%) accounted for over 94% of the total ASVs in all treatments, making them the dominant fungal phyla in the rhizosphere of *P. tenuifolia* (**Figure 5A**). Ascomycota are the dominant fungal species in global soil, followed by Basidiomycota (Egidi et al., 2019). The dominant genera in each treatment were *Alternaria* (RA: 7.30%-15.57%), *Solicoccozyma* (4.81%-7.72%), *Didymella* (3.28-6.78%), *Mortierella* (3.43-8.26%), *Fusarium* (4.54-7.58%), *Neocosmospora* (2.50-4.86%), *Cladosporium* (1.70-4.65%) and *Papiliotrema* (1.88-3.92%), respectively (**Figure 5B**). The RAs of *Mortierella* were significantly increased in SMI2 (*p*<0.05). *Mortierella* has been reported as a phosphate-solubilizing fungus with the ability to mobilize P from insoluble forms and protect crops from pathogen invasion (Sharma et al., 2013; Alori et al., 2017; Ozimek and Hanaka, 2021; Ning et al., 2022). The abundance of the genus *Cladosporium*, which contains various plant and human pathogenic fungi, decreased in MFs, with reductions of 33.33% in MF1 and 57.93% in MF2.

According to LEfSe analysis, there were also differences in fungal biomarkers of *P. tenuifolia* rhizosphere across different groups (**Figures 5C, D**). *Cladorrhinum*, which was enriched in CF, was found to be detrimental to plants or humans. However, this negative effect was significantly inhibited in BOF groups, suggesting that the application of BOFs reduced the RAs of certain harmful fungal genera in *P. tenuifolia* rhizosphere soil. Furthermore, different groups also demonstrated enrichment of beneficial fungal genera in the soil. For instance, *Aureobasidium* and *Purpureocillium* enriched in PMI1 group, which can serve as plant growth promoters and biological control agents (Bao et al., 2022; Rensink et al., 2024). The biomarker MF1, *Hannaella*, exhibits the biocontrol effect, and *Hannaella* sp. capable of inducing plant disease resistance (Lin et al., 2022; Yang et al., 2023).

### 3.5 Correlation analysis of plant traits, soil properties, and rhizosphere microbial communities

Pearson correlation analysis were conducted to investigate the relationship among plant traits (growth parameters and bioactive components contents) and soil properties (**Supplementary Figure S2**). A significant positive correlation was observed between the content of DISS and both underground biomass and root diameter of *P. tenuifolia* (*p* < 0.05). Studies on the active components and medicinal specifications of *P. tenuifolia* have shown that the content of DISS was positively correlated with root length but highly negatively correlated with plant height (Zhang et al., 2022). Saponins in *P. tenuifolia* are mainly distributed in the root parenchyma and stored within the secondary phloem (Teng et al., 2009). This may explain the significant correlation between the active components content in *P. tenuifolia* and its underground parts, especially the biomass of phloem. Based on our research findings, it can be inferred that the application of microalgae may enhance the medicinal quality by increasing the underground biomass of *P. tenuifolia*.

**Figure 6:**
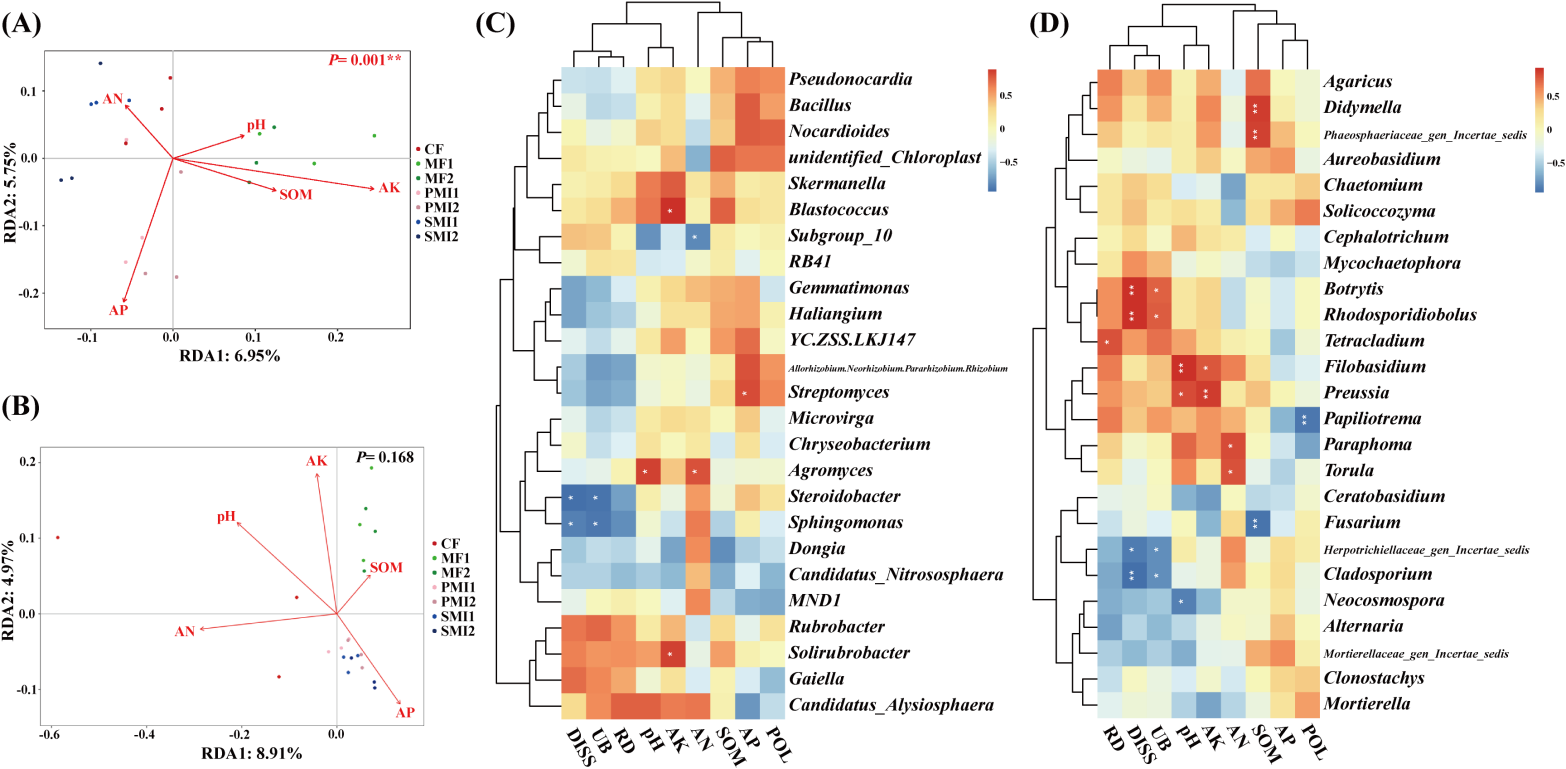
Relationship among plant traits, soil properties, and rhizosphere microbial communities. Redundancy analysis (RDA) reveals relative importance of soil properties on the rhizosphere bacterial community **(A)** and fungal community **(B)**. Heatmap analysis of the correlation among the composition of rhizosphere bacteria **(C)** and fungi **(D)** at the genus level, soil properties and plant traits. *, *p* < 0.05; **, *p* < 0.01. SOM, soil organic matter; AN, alkali-hydrolyzable nitrogen; AK, available potassium; AP, available phosphorus; POL, polygalaxanthone III; DISS, 3,6’-disinapoylsucrose. CF, PMI1, PMI2, SMI1, SMI2, MF1, and MF2 were as defined in the footnote to **Figure 1**.

The effects of soil properties on the rhizosphere microbial community of *P. tenuifolia* upon fertilizer application were investigated by RDA analysis. It was found that soil properties had more significant effects on rhizosphere bacterial structure than fungi (**Figures 6A, B**). AP and AK significantly influenced soil bacterial community composition, while pH, AN and AK had a remarkable impact on soil fungal community composition (**Table 2**). In conclusion, pH and available nutrients were all important factors influencing the structure of rhizosphere microbial communities. Further analysis revealed a close association between certain rhizosphere microorganisms and available soil nutrients (**Figures 6C, D**). The content of AP showed a significant positive correlation with *Streptomyces* (*p* < 0.05), which had the highest RAs in PMIs and were identified as biomarkers for PMI2. These microorganisms are capable of solubilizing nutrients sequestered in the crystalline lattice of soil mineral fraction through the secretion of low molecular weight organic acids such as gluconic acid, citric acid, succinic acid, and oxalic acid (Rajput et al., 2013; Timofeeva et al., 2022). As a result, the AP content of PMI1 and PMI2 were significantly increased (*p* < 0.05). Consequently, the application of PMIs promoted the growth of *P. tenuifolia* by recruiting *Streptomyces* to enhance phosphorus availability in soil. The content of pH and AN exhibited positive correlation with the RAs of *Agromyces*, which related to nitrogen-converting. Some *Agromyces* sp. have been identified to possess the nitrogen fixation gene (*nifH*) and have been experimentally verified to exhibit nitrogen-fixing ability (Zhou et al., 2014). AK was significantly positively correlated with *Blastococcus* and *Solirubrobacter* (*p* < 0.05). *Solirubrobacter* acts as a PGPR and also facilitates the mobilization of potentially toxic elements (Jiang et al., 2023; Cebekhulu et al., 2024). In short, using bio-organic fertilizer as a partial substitution for chemical fertilizer can recruit beneficial microorganisms and improve soil fertility, with complex interactions observed between rhizosphere microbial activity and soil properties.

**Table 2:** P value of correlation between microbial community composition and environmental factors.

Parameters	Bacterial composition		Fungal composition	
	r^2^	*p*	r^2^	*p*
pH	0.1020	0.372	0.4110	0.008**
SOM	0.2143	0.109	0.0647	0.517
AN	0.1292	0.284	0.5194	0.002**
AP	0.6935	0.001***	0.2552	0.060
AK	0.7168	0.001***	0.2755	0.004**

**, *p* < 0.01; ***, *p* < 0.001. SOM, AN, AP and AK were as defined in the footnote to **Figure 6**.

According to the Pearson test between plant traits and microbial community, underground biomass and DISS content were positively correlated with the RA of Chloroflexi (*p* < 0.01) and negatively correlated with the RA of Proteobacteria (*p* < 0.05) (**Supplementary Figure S3**). Rhizosphere microbial communities exhibited a response to reduced chemical fertilizer application and the utilization of BOFs by promoting the proliferation of oligotrophs (e.g., some Chloroflexi) while suppressing copiotrophs (e.g., some Proteobacteria) (Dai et al., 2018). Oligotrophs such as Chloroflexi exhibited a pronounced substrate affinity and preferentially decompose resistant carbon (Hug et al., 2013; Banerjee et al., 2016). Chloroflexi plays a central role in the symbiotic relationships between soil bacteria, fungi, and plants. Specifically, Chloroflexi can fix CO_2_ and convert inorganic carbon into biodegradable organic matter, and it also acts as producers of nutrients such as phosphorus and nitrogen (Narsing Rao et al., 2022; Freches and Fradinho, 2024). In this study, Chloroflexi exhibited the highest RAs in MFs, showing a significant increase of 31.06% and 38.27% compared to CF in MF1 and MF2, respectively. Additionally, SOM content in MF1 was significantly higher than CF (*p* < 0.05). Therefore, the application of microalgae may positively stimulate specific species within Chloroflexi, thereby improving soil carbon cycling, enhancing soil fertility, promoting nutrient absorption by plants, and ultimately increasing biomass accumulation and bioactive component production in *P. tenuifolia*.

## 4 Conclusion

This study focused on *P. tenuifolia*, assessing the impact of substituting chemical fertilizer with BOFs on its underground growth, bioactive component contents, soil properties, rhizosphere bacterial and fungal communities. The objective is to determine how BOF application affects *P. tenuifolia*’s growth, quality, soil fertility, and the rhizosphere microenvironment. The results showed that BOFs maintained the quality and yield of *P. tenuifolia* while reducing chemical fertilizer application by 20% and 40%. Among them, MF not only exhibited the greatest growth-promoting effect but also significantly enhanced the accumulation of bioactive components in *P. tenuifolia*. Substituting 40% chemical fertilizer with microalgae resulted in a 29.30% increase in underground biomass and a 19.72% increase in 3,6’-disinapoylsucrose content. microalgae partially substituting chemical fertilizer also significantly altered the composition of rhizosphere microbial communities, with a greater impact on bacterial community rather than fungal community. MF and PMI partially substituting for chemical fertilizer enhanced soil fertility by increasing organic matter and available phosphorus levels, respectively. Cholorflexi exhibited the highest relative abundances in MFs, and correlation analysis revealed a positive association between plant traits and Cholorflexi, indicating that microalgae partial substitution for chemical fertilizer may stimulate Chloroflexi species associated with carbon cycling, thereby enhancing both growth and quality of *P. tenuifolia*. Moreover, *Streptomyces* had the highest relative abundances in PMIs and was identified as biomarker for PMI2, and correlation analysis also revealed a positive association between available phosphorus content and *Streptomyces*, indicating that PMI may recruit *Streptomyces* to increase the soil available phosphorus content without compromising the underground growth and bioactive component contents of *P. tenuifolia*. In conclusion, these findings underscore the potential of microalgae and microbial inoculant as chemical fertilizer substitutes to enhance the growth and quality *P. tenuifolia* by improving soil microorganisms. This study provides a theoretical foundation for the utilization of microalgae and microbial inoculants as partial substitutes for chemical fertilizer to enhance productivity and improve soil quality in medicinal plants.

## Data Availability

The original contributions presented in the study are publicly available. This data can be found here: https://www.ncbi.nlm.nih.gov/bioproject/PRJNA1206537.
