# Supplementary material for: Microalgae and microbial inoculant as partial substitutes for chemical fertilizer enhance Polygala tenuifolia yield and quality by improving soil microorganisms
Source: Front Plant Sci. 2025 Jan 16;15:1499966. doi: 10.3389/fpls.2024.1499966 (PMC11779722; doi:10.3389/fpls.2024.1499966)
Supplement: Supplementary file 1 [file DataSheet1.pdf]

Supplementary Material

1 Supplementary Figures and Tables

1.1 Supplementary Figures

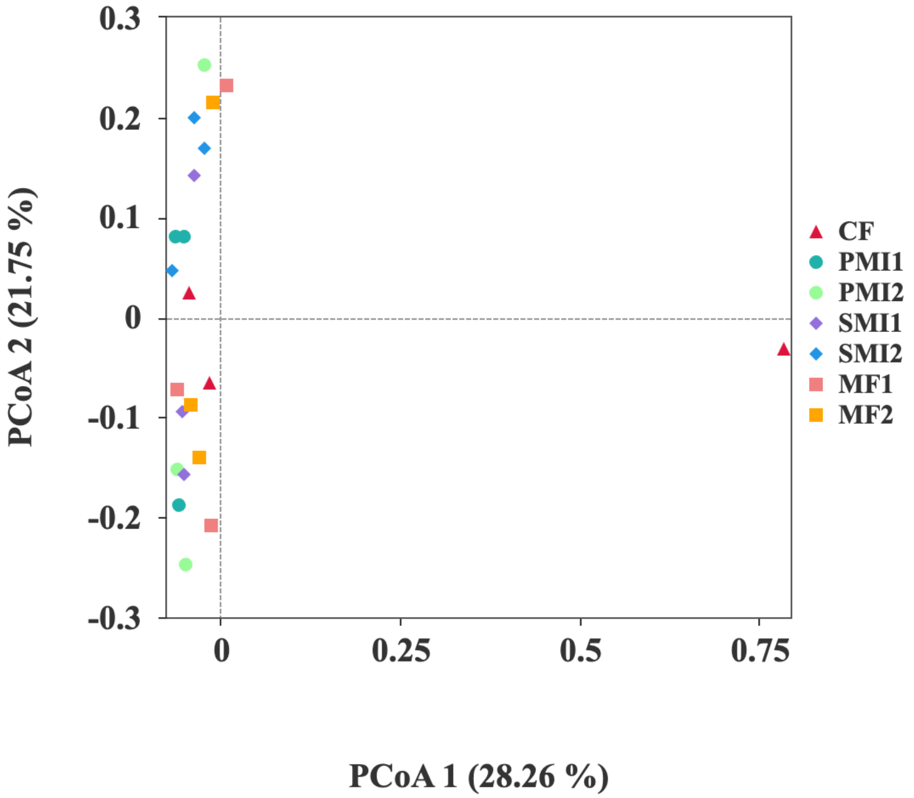

Supplementary Figure 1. Principal coordinate analysis (PCoA) plot based on the rhizosphere fungal community composition. CF, chemical fertilizer; PMI1, -20% chemical fertilizer + polyglutamic acid microbial inoculant; PMI2, -40% chemical fertilizer + polyglutamic acid microbial inoculant; SMI1, -20% chemical fertilizer + solid microbial inoculant; SMI2, -40% chemical fertilizer + solid microbial

inoculant; MF1, -20% chemical fertilizer + microalgae fertilizer; MF2, -40% chemical fertilizer + microalgae fertilizer.

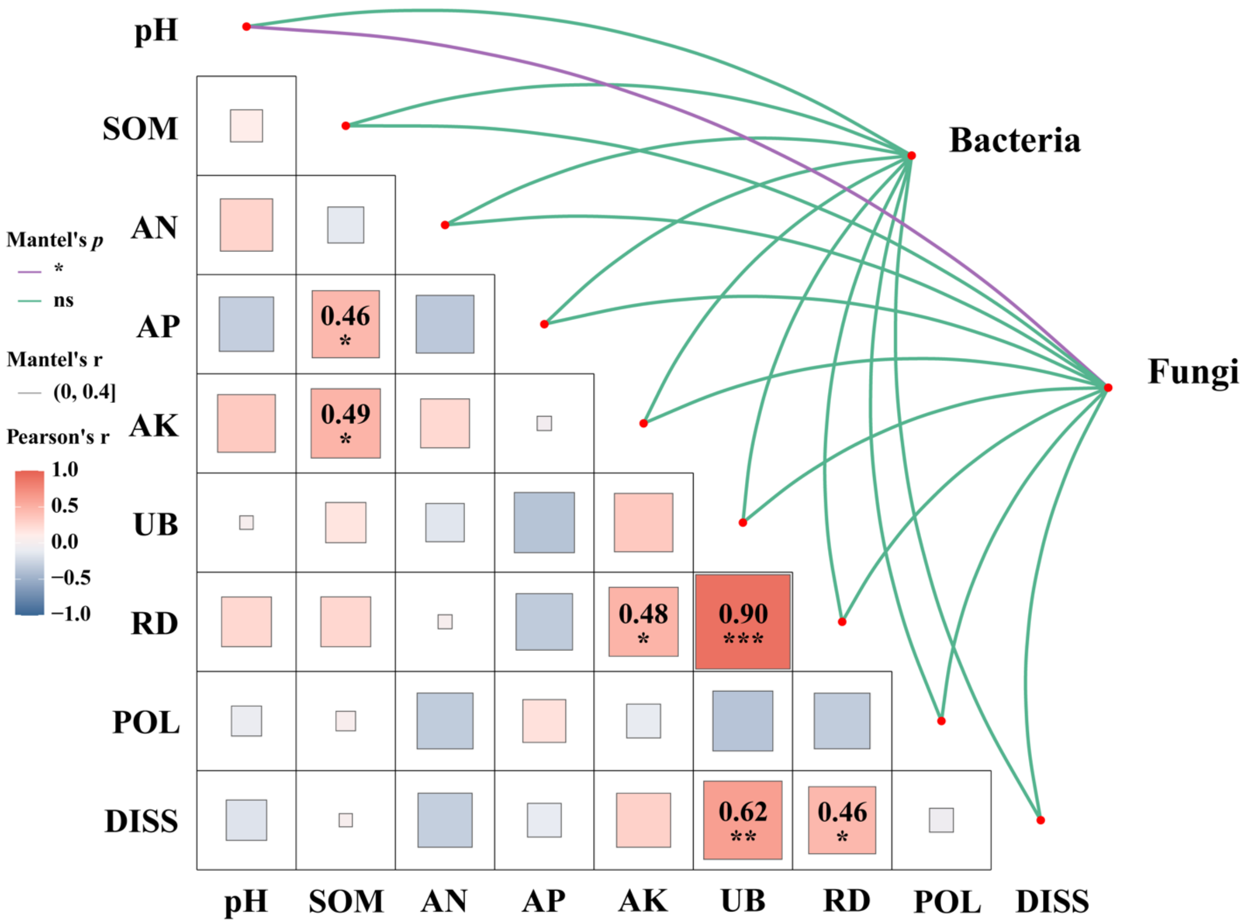

Supplementary Figure 2. Pearson correlation analysis of soil physicochemical properties and *P. tenuifolia* plant traits (growth indicators, bioactive components content), and the Mantel test analysis of microbial communities, plant traits and soil properties. SOM, soil organic matter; AN, soil alkali-

hydrolyzable nitrogen; AP, available phosphorus; AK, available potassium; POL, polygalaxanthone III; DISS, 3,6’-disinapoylsucrose. *, $p < 0.05$; **, $p < 0.01$; ***, $p < 0.001$.

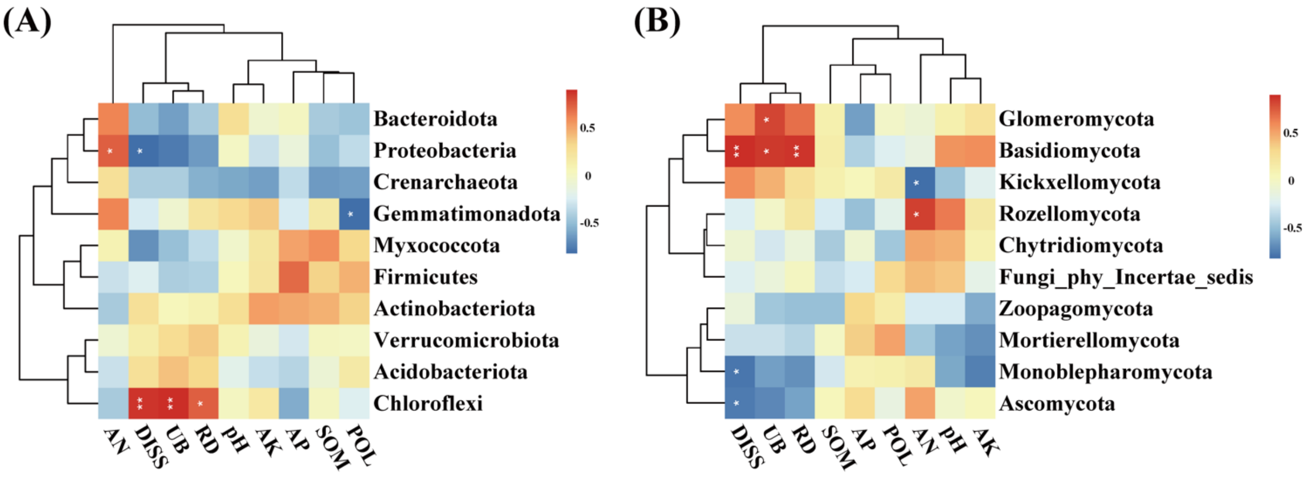

Supplementary Figure 3. Heatmap analysis of the correlation among the composition of rhizosphere bacteria (A) and fungi (B) at the phylum level, soil properties and plant traits. CF, PMI1, PMI2, SMI1, SMI2, MF1, and MF2 were as defined in the footnote to Fig S1. SOM, AN, AK, AP, POL, and DISS were as defined in the footnote to Fig S2. *, $p < 0.05$; **, $p < 0.01$; ***, $p < 0.001$.

1.2 Supplementary Tables

Supplementary Table 1. Richness and diversity indices of bacteria and fungi

	Treatments	Chao1	Shannon
Bacteria	CF	3519.79±76.31^{a}	10.79±0.04^{a}
	PMI1	3333.38±207.95^{a}	10.63±0.10^{a}
	PMI2	3322.86±172.69^{a}	10.59±0.16^{a}
	SMI1	3437.00±323.44^{a}	10.69±0.14^{a}
	SMI2	3411.54±265.41^{a}	10.76±0.03^{a}
	MF1	3243.65±275.34^{a}	10.72±0.15^{a}
	MF2	3285.43±228.62^{a}	10.67±0.13^{a}
Fungi	CF	352.42±25.89^{a}	6.04±0.69^{a}
	PMI1	317.03±32.07^{a}	5.49±0.56^{a}

PMI2	315.61±20.34[a]	5.25±0.34[a]
SMI1	315.64±22.02[a]	5.56±0.35[a]
SMI2	348.77±34.01[a]	5.81±0.21[a]
MF1	353.60±45.17[a]	5.71±0.35[a]
MF2	330.64±32.94[a]	5.41±0.36[a]

CF, PMI1, PMI2, SMI1, SMI2, MF1, and MF2 were as defined in the footnote to Fig S1. Values within each column followed by different lowercase letters are significant at $p < 0.05$.
